# Enhancing the Use of Argos Satellite Data for Home Range and Long Distance Migration Studies of Marine Animals

**DOI:** 10.1371/journal.pone.0040713

**Published:** 2012-07-12

**Authors:** Xavier Hoenner, Scott D. Whiting, Mark A. Hindell, Clive R. McMahon

**Affiliations:** 1 Research Institute for Environment and Livelihoods, Charles Darwin University, Darwin, Northern Territory, Australia; 2 Department of Natural Resources, Environment, the Arts and Sport, Northern Territory Government, Darwin, Northern Territory, Australia; 3 Department of Environment and Conservation, Government of Western Australia, Kensington, Western Australia, Australia; 4 Institute for Marine and Antarctic Studies, University of Tasmania, Hobart, Tasmania, Australia; University of Wales Swansea, United Kingdom

## Abstract

Accurately quantifying animals’ spatial utilisation is critical for conservation, but has long remained an elusive goal due to technological impediments. The Argos telemetry system has been extensively used to remotely track marine animals, however location estimates are characterised by substantial spatial error. State-space models (SSM) constitute a robust statistical approach to refine Argos tracking data by accounting for observation errors and stochasticity in animal movement. Despite their wide use in ecology, few studies have thoroughly quantified the error associated with SSM predicted locations and no research has assessed their validity for describing animal movement behaviour. We compared home ranges and migratory pathways of seven hawksbill sea turtles (*Eretmochelys imbricata*) estimated from (a) highly accurate Fastloc GPS data and (b) locations computed using common Argos data analytical approaches. Argos 68^th^ percentile error was <1 km for LC 1, 2, and 3 while markedly less accurate (>4 km) for LC ≤0. Argos error structure was highly longitudinally skewed and was, for all LC, adequately modelled by a Student’s *t* distribution. Both habitat use and migration routes were best recreated using SSM locations post-processed by re-adding good Argos positions (LC 1, 2 and 3) and filtering terrestrial points (mean distance to migratory tracks ± SD = 2.2±2.4 km; mean home range overlap and error ratio  = 92.2% and 285.6 respectively). This parsimonious and objective statistical procedure however still markedly overestimated true home range sizes, especially for animals exhibiting restricted movements. Post-processing SSM locations nonetheless constitutes the best analytical technique for remotely sensed Argos tracking data and we therefore recommend using this approach to rework historical Argos datasets for better estimation of animal spatial utilisation for research and evidence-based conservation purposes.

## Introduction

Global economic development puts increasing pressure on terrestrial and marine ecosystems for the exploitation of natural resources. Commercial activities (e.g. fishing, mining and oiling exploitation) threaten to deteriorate animal habitats and therefore put their survival at risk [1], [2], [3]. To mitigate human impact and adequately delimitate protected areas, determining the distribution of wildlife is paramount. Quantifying habitat use is similarly vital to understand animals’ biophysical requirements (e.g. nutrition, reproduction) and further predict areas of ecological significance [4], [5], [6]. Breeding and foraging grounds are especially important for conservation as those areas constitute crucial habitats in animals’ lifecycles. Reproductive migration also represents a key phase during which animals are exposed to various anthropogenic threats over long distances. Estimating home ranges and migratory corridors is however nontrivial and limited by the accuracy of the tracking technique and the analytical methods used to estimate animal position. Such assessment becomes even more technically challenging when researching migratory animals, such as marine turtles, that range over thousands of kilometres [7], [8].

Satellite telemetry is now the most commonly used technique to study long-distance migrants as they can be tracked remotely and regularly for many months [9], [10], [11], [12], [13]. Two different systems exist. Service Argos uses the Doppler shift in transmitted frequencies to estimate animal location [14]. Positions are subsequently classified into one of seven location classes (LC 3, 2, 1, 0, A, B, and Z) and have a 68^th^ percentile spatial error ranging from 0.5 (LC 3) to 10 km (LC B) [15], [16], [17]. However, as air breathing marine animals commonly surface only briefly, extended transmission opportunities are rare, resulting in high proportions of locations with high spatial errors (LC 0, A and B) [18], [19]. The Fastloc GPS system overcomes this impediment by having fast acquisition times (<100 ms) and uses the Global Positioning System (GPS) to compute animal location with higher accuracy (95^th^ percentile error <140 m) [20]. Lower measurement errors, combined with more frequent fixes [21], [22], has enabled researchers to quantify animal movement behaviour at finer scales [23], [24], [25] and calculate more realistic habitat use maps resulting in improved management recommendations, including underpinning the designs of protected areas [26], [27], [28]. Furthermore, tracking animals simultaneously with both systems enables the quantification of the error associated with each Argos LC, which is paramount for enhancing the accuracy of Argos location estimates by incorporating error structures into mathematical models [15], [16], [17], [29]. As Argos datasets have been collected for over three decades, using correcting algorithms to rework historical datasets is a necessary step to obtain better estimates of animal habitat utilisation and thus potentially avoid the need to repeat studies with newer technology.

Although commonly applied to remotely sensed movement data, *ad-hoc* heuristic methods for refining Argos location estimates (e.g. speed filters) are subjective and discard substantial amounts of potentially valuable data [10], [30], [31]. A more parsimonious approach consists in fitting state-space models (SSMs) to Argos locations [32], [33]. SSMs separately account for Argos LC error structure and stochasticity in animal movement using behavioural correlated random walk models [34], [35], [36]. Irregular, non-Gaussian error distributions are incorporated into this complex statistical framework using Markov Chain Monte Carlo (MCMC) estimation methods. Albeit computer intensive, this Bayesian statistical framework does not remove extreme observations as do other likelihood-based methods (e.g. Kalman filters) [37], [38]. Despite their robustness and wide use in ecological research, few studies have yet tested the accuracy of SSM predicted locations, especially for subsequent geospatial analyses estimating habitat utilisation [38], [39].

Hawksbill turtles (*Eretmochelys imbricata*, Linnaeus 1766) are migratory marine animals distributed circumtropically (Witzell 1983, Márquez 1990, Leon & Bjorndal 2002). Upon reaching sexual maturity, individuals select a foraging ground where they exhibit high site fidelity [40], [41]. Episodically though, adults migrate to the vicinity of their natal site to reproduce. Females breed every two to six years, laying several clutches of eggs at a two to three week intervals [42], [43], [44], [45]. Between nesting events, *i.e.* the inter-nesting period, females commonly inhabit the waters surrounding their nesting sites [43], [45], [46]. Once the nesting season is over, hawksbill turtles undertake post-nesting migrations to return to their feeding sites [41]. Tracking hawksbill turtles from their breeding site consequently provides information on their inter-nesting, migratory and foraging behaviour. Using satellite tracking data from seven hawksbill turtles, this study successfully quantified the spatial error associated with commonly used Argos statistical processing methods and identified the analytical approach best enhancing the accuracy of location estimates and home ranges. We additionally complemented this critical technical assessment by thoroughly examining Argos location error structure to examine the consistency of our data with previous marine vertebrate tracking studies and for future incorporation into complex correcting algorithms.

## Materials and Methods

### Ethics Statement

All necessary permits were obtained for the described field studies. The animal use protocol for this research was reviewed and approved by the Animal Ethics Committee of Charles Darwin University and met the requirements of the Australian Code of Practice for the Care and Use of Animals for Scientific Purposes (1997) and the Northern Territory Animal Welfare Act (1999) (Project Reference No A04005). A permit to undertake scientific research on wildlife was also obtained from the Northern Territory Government (Marine Biodiversity Group - Department of Natural Resources, Environment, the Arts and Sport) - Primary Holder: Scott Whiting; Nominees on Permit: Xavier Hoenner and Elisabeth Dethmers (Reference No 39239). The Groote Eylandt archipelago constitutes an Indigenous Protected Area and is Indigenous owned. Permits and permission to carry research on Indigenous land was obtained by the Anindilyakwa Land Council.

### Attachment Details

We attached, after oviposition, a Satellite Relay Data Logger (SRDLs, Sea Mammal Research Unit, St. Andrews, U.K.) on each of seven adult female hawksbill turtles nesting on Groote Eylandt, northern Australia (13°58 S, 136°35 E). We mounted SRDLs onto wedges to maximise communication efficiency between tags and satellites (base  = 102 mm, width  = 5 mm, height  = 30 mm, hypotenuse  = 106 mm, slope  = 16°) [47]. Using quick-setting two-part epoxy resin (Sika AnchorFix®-3+, Sika Australia Pty Ltd), we glued transmitters and wedges onto the flat part between the two anterior central scutes of the turtles’ shell. As the satellite transmitters we used were hydrodynamic and represented less than 1.5% of hawksbill turtles’ weight, we presumed that they had minimal effect on individual behaviour (SRDL  = 700 g, average weight of adult female hawksbill turtles  = 48.7 kg) [48], [49]. We released all tagged animals unharmed when the epoxy had totally cured. SRDLs used the Service Argos telemetry system to transmit Fastloc GPS data, subsequently providing two sets of locations: Argos and Fastloc GPS [18], [29].

### Argos Location Class: Error Structure

We first examined the error structure of Argos LC following methods in Costa et al. (2010) [15]. We first isolated Argos locations obtained within five minutes of a GPS position. We then estimated animal “true” position at the time of the Argos uplink by linearly interpolating neighbouring GPS coordinates. Following this procedure, we computed the error distance between Argos locations and “true” animal positions and examined the latitudinal and longitudinal components of error. To investigate Argos error distribution for each LC, the latitudinal and longitudinal error components were subsequently fitted to a *t* distribution using a maximum likelihood approach. The *t* distribution allows for robust incorporation of outliers and it best modelled all Argos LC estimation errors except for LC 3 estimates [35]. For better knowledge of Argos error distributions, we produced joint log-likelihood surface plots with 95% confidence regions for the two parameters influencing the *t* distribution (*i.e.* the scale parameter τ and the degree of freedom ν). As the Gaussian distribution is a special case of the *t* distribution when ν **→ ∞**, the shape of the 95% confidence region indicates the suitability of the *t* distribution to model each Argos LC error structure. We subsequently compiled maximum likelihood estimates of τ and ν for comparison with other studies.

**Figure 1:**
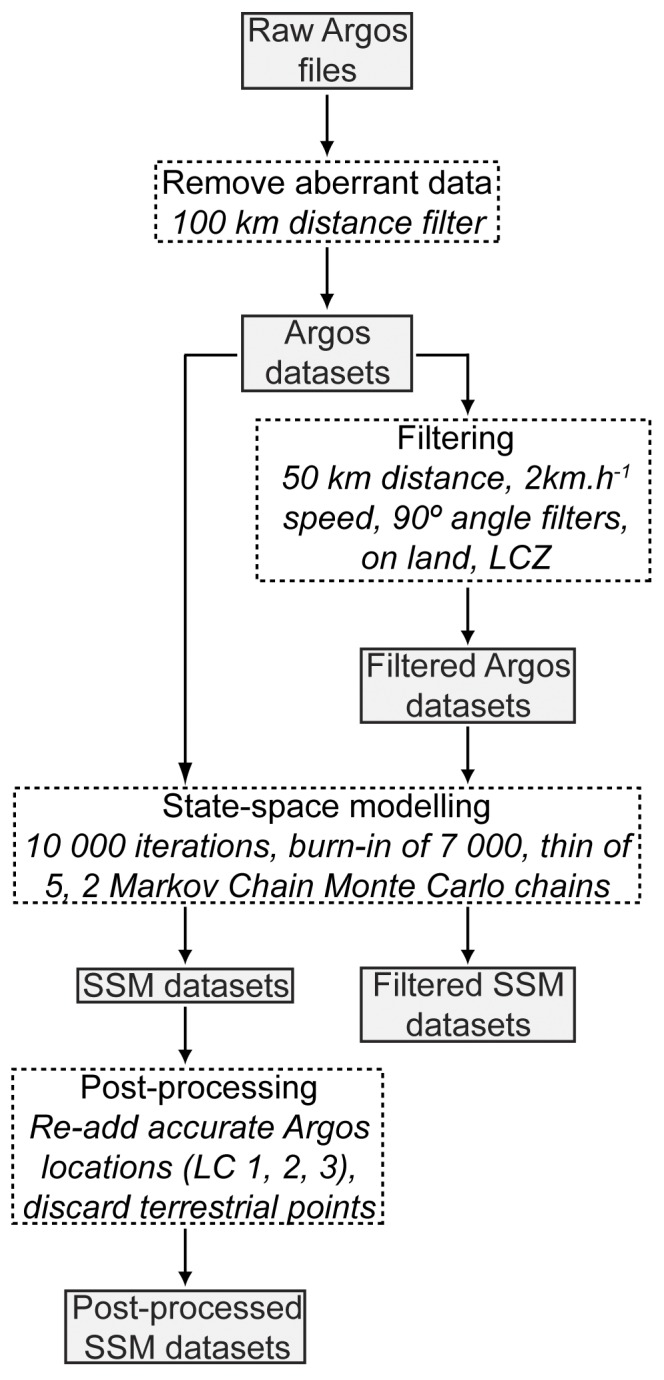
Flow diagram showing the different approaches used to obtain our five Argos-derived datasets. Data transformation procedures are indicated within dashed line boxes, datasets are indicated within solid line boxes.

### Data Pre-processing

For each individual we compiled a Fastloc GPS and several Argos-based datasets through discrete processing approaches (Figure 1). We first applied a 100 km distance filter to raw Argos and Fastloc GPS datasets to discard the most erroneous locations. This procedure removed aberrant Fastloc GPS positions that were not isolated with standard filtering algorithms (i.e. location estimates derived from fewer than five satellites or with residual errors ≥30) [29]. Argos datasets were then used to produce (i) filtered Argos and (ii) state-space model (SSM) datasets. We computed filtered Argos datasets by combining a set of distance, speed, angle and location class filters commonly used in tracking experiments and by removing terrestrial fixes using data from the Australian Bathymetry and Topography Grid [50]. We adopted the following filter thresholds as they produced biologically relevant movement patterns while minimizing information loss and were consistent with previous studies on hawksbill turtles: 50 km, 2 km.h^−1^, 90°, LC Z [51], [52]. A state-space analysis was applied to both the Argos and filtered Argos datasets using the hierarchical two state-switching correlated random walk model in R and WinBUGS [37], [53], [54]. To enhance the accuracy of predicted locations, we grouped individuals with similar data collection frequency [36], [55] and we adopted the following parameters to run our model: 10000 iterations, a burn-in of 7000, a thin of 5 and two MCMC chains. We then assessed convergence in WinBUGS by calculating the Gelman-Rubin convergence statistic and through trace plot examination for “mixing” and stationarity [54]. Through this procedure we obtained a SSM and filtered SSM dataset for each individual. Finally, we attempted to further refine SSM datasets by discarding terrestrial points and re-adding high quality Argos locations (LC 1, 2, and 3). Prior to geospatial analysis, we distinguished the inter-nesting, migration and foraging phases for each individual and each dataset by successively mapping animal positions through time in R, using standard habitat discrimination criteria for marine turtles [56], [57].

**Figure 2:**
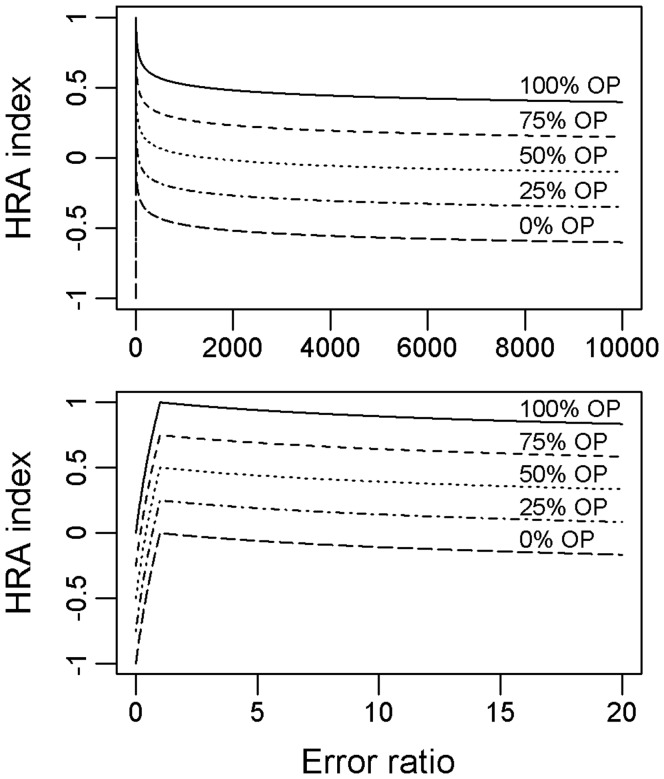
Home Range Accuracy (HRA) index as a function of the overlaying percentage (OP) and error ratio (ER). The top panel represents the evolution of the HRA index for ERs comprised between 0 and 10 000. The bottom panel highlights the smooth join for ER  = 1.

### Home Range Analyses

To assess habitat use during the inter-nesting and foraging periods, we calculated the 50% and 95% utilisation distribution (UD) using the fixed Kernel Density Estimation (KDE) method derived from least-squares cross-validation bandwidths [58], [59]. The 50% UD area represents an animal’s core area of activity while the 95% area determines its overall home range [28], [60]. As female hawksbill turtles frequented a common marine area during the inter-nesting period, we computed the combined utilisation distribution (UD) for our seven breeding animals by aggregating individuals’ locations. We used random sampling to account for inter-individual differences in numbers of inter-nesting locations and computed UD for 10 000 bootstrap iterations to explore possible home range sizes and shapes.

### Comparing Home Ranges

Argos and GPS home ranges were compared by estimating the overlaying percentage (OP) and error ratio (ER). These two parameters respectively quantify the percentage of overlap between the GPS and Argos-derived home ranges and examine their size ratio using the following formula [61], [62]:
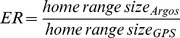



**Figure 3:**
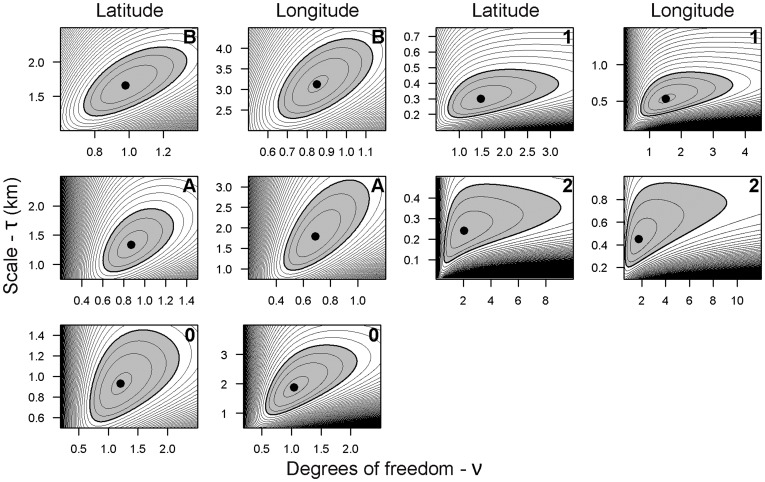
Joint log-likelihood surface plots for *t* distribution parameters τ and ν, for the longitude and latitude components of error. Argos location classes are indicated in the lower right corner of each panel. Maximum likelihood estimates are represented by filled circles. The 95% confidence region on each panel is indicated in gray and delimitated by a thick black line. The contour interval is -1 with log-likelihood values decreasing from the maximum likelihood point estimate.

We then designed a Home Range Accuracy (HRA) index for automatic and objective discrimination of home range estimates. This HRA index, built as a smooth joining algorithm, varies between 1 (OP  = 100%, ER  = 1) and -1 (no overlap, ER → 0).

(1) For ER ≤1:
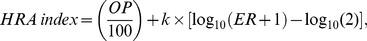



k  =  log_0.5_(10).

(2) For ER >1:
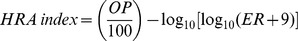



Because encompassing animal habitats is often the primary objective of conservation-oriented ecological studies, we assigned more importance on the OP than the ER term and penalised home range size underestimation more severely than overestimation by applying a steeper decrease for ER ≤1 (Figure 2). The value of k was determined so that the second term in eq. (1) is equal to -1 for ER  = 0. Similarly, log_10_(2) and 9 allow for smooth joining of the two equations for ER  = 1.

To further explore the relationship between Argos and GPS home range size, we fitted a set of polynomial generalised linear models to those data (*i.e.* null, linear, quadratic, cubic and quartic model) and evaluated the relative strength of evidence of each candidate model using multi-model inference, based on information theoretic [63]. More specifically we used the Akaike’s Information Criterion corrected for small sample size (AIC_c_) and its associated weight wAIC_c_.

### Assessing Migratory Pathways

We additionally assessed the error associated with Argos migratory pathways by computing the minimum distance between location estimates and interpolated Fastloc GPS data. Assuming constant speed and linear paths between locations, we linearly interpolated neighbouring GPS positions to obtain one point every 200 metres, thereby recreating animals’ “true” migratory tracks.

**Table 1:** Comparison of the 68^th^ percentile spatial error associated with each Argos location class from different studies (in km).

Source	Methods	LC3	LC2	LC1	LC0	LCA	LCB	N
ARGOS	Theoretical	0.15	0.35	1.00	–	–	–	
This study	On animals, at sea	0.51	0.67	1.02	4.15	10.19	9.24	506
Costa et al. (2010)	On animals, at sea	0.49	1.01	1.20	4.18	6.185	10.28	1105
Hazel et al. (2009)	On animals, at sea	0.48	0.79	1.43	5.18	8.07	11.48	168

## Results

**Table 2:** Maximum likelihood estimates and standard errors for *t* distribution parameters τ and ν for longitudinal and latitudinal components of Argos error.

LC	Longitude		Latitude		N
	τ_lon_ (SE)	ν_lon_ (SE)	τ_lat_ (SE)	ν_lat_ (SE)	
B	3.134 (0.379)	0.854 (0.094)	1.659 (0.206)	0.982 (0.121)	261
A	1.789 (0.440)	0.690 (0.124)	1.328 (0.236)	0.870 (0.146)	104
0	1.877 (0.457)	1.054 (0.269)	0.926 (0.170)	1.211 (0.275)	66
1	0.534 (0.115)	1.528 (0.482)	0.301 (0.063)	1.478 (0.448)	43
2	0.452 (0.145)	1.752 (0.920)	0.239 (0.073)	2.048 (1.162)	18

N represents the number of Argos locations of each location class (LC) obtained within five minutes of a GPS uplink.

Hawksbill turtles only relayed a low proportion of LC >0 positions (mean ± SD  = 9.5±7.3%) (Table S1). On average GPS locations were transmitted more frequently but for slightly shorter time periods than Argos positions (mean daily transmission frequency  = 3.6±0.6 vs. 2.3±0.9 locations, mean tracking duration  = 171.9±125.4 vs. 203.2±137.6 days respectively) (Table S1). Argos location 68^th^ percentile errors were relatively low for LC 1, 2 and 3 (0.51, 0.67 and 1.02 km respectively) (Table 1). Those errors were similar to previous studies but larger than Argos theoretical estimates (Table 1). LC A and B locations showed similar errors of about 10 km while LC 0 positions were associated with a 4.2 km 68^th^ percentile error (Table 1). The 95% confidence regions on joint log-likelihood surface plots indicate that the observational error structure associated with Argos location estimates of each LC follows a Student’s *t* distribution (Figure 3). Although insufficient sample size prevented distribution fitting of Argos LC 3 positional errors, we found that the 95% confidence region upper limit for the degree of freedom (ν) increased with the quality of Argos LC (Figure 3). Maximum likelihood estimates of the scale parameter τ showed larger longitudinal than latitudinal components of error for all LCs (Table 2).

**Figure 4:**
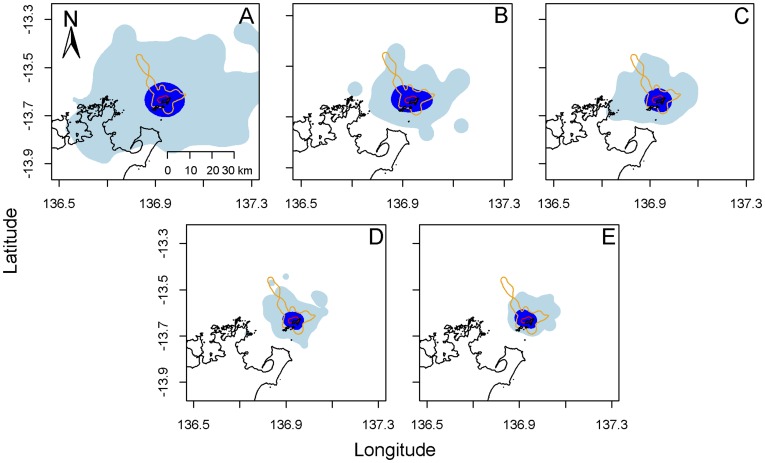
Combined 50% and 95% utilisation distribution (UD) contour polygon calculated for each Argos-based approach compared to GPS estimates. The core GPS area (50% UD) and overall home range (95% UD) are delimitated by a red and orange line respectively. Argos-based 50 and 95% UD polygons are coloured in dark and light blue respectively. (A) Argos, (B) filtered Argos, (C) SSM, (D) post-processed SSM, (E) filtered SSM.

The combined core inter-nesting area (50% UD) was best estimated using post-processed SSM locations (HRA index  = 0.943) (Table 3). All processing approaches produced 50% UD polygon fully encompassing the core GPS 50% UD polygon (100% overlap) (Figure 4). Argos and filtered Argos datasets produced core areas twice the size of SSM-derived locations (ER >14.0 against ER <7.0 respectively) (Table 3). Post-processing SSM locations improved home range size estimates by 72.7% and 30.4% respectively compared to Argos and SSM estimates (Table 3). The combined 95% UD analysis, on the other hand, identified the filtered Argos locations as best recreating the overall GPS area (mean ER  = 6.8, mean OP  = 97.2%), producing size estimates 3.4 times more accurate than Argos-based home range while only inducing a 2.8% loss in overlap (HRA index  = 0.893) (Table 3, Figure 4B). SSM approaches produced the lowest ERs (ER <5.0) but failed to encompass completely the overall combined inter-nesting area (OP<90.0%) (Table 3, Figure 4C, D, and E). Contrarily to our 50% UD analysis, we observed a parallel decrease of the ER and OP parameters for increasing complexity of data processing.

**Table 3:** Mean HRA index (error ratio/overlaying percentage) associated to individual and combined inter-nesting home range estimates using Argos-derived locations.

	Combined home range		Individual home range	
	50% UD	95% UD	50% UD	95% UD
Argos	0.846 (17.6/100)	0.822 (23.1/100)	0.573 (2712.0/90.7)	0.598 (2948.6/97.0)
Filtered Argos	0.865 (14.2/100)	**0.893** **(6.8/97.2)**	0.595 (2224.8/88.1)	0.677 (867.7/95.4)
SSM	0.920 (6.9/100)	0.843 (4.9/90.0)	0.627 (648.0/90.1)	0.688 (496.4/95.0)
Post-processed SSM	**0.943** **(4.8/100)**	0.832 (3.3/86.9)	**0.657 (376.0/89.3)**	**0.718 (195.1/95.0)**
Filtered SSM	0.938 (5.3/100)	0.798 (2.4/82.2)	0.591 (348.5/82.1)	0.653 (177.0/90.4)

50% and 95% UD refer to the core area of activity and overall home range respectively. Values in bold highlight the best refining approach for each Argos-based home range estimate.

**Figure 5:**
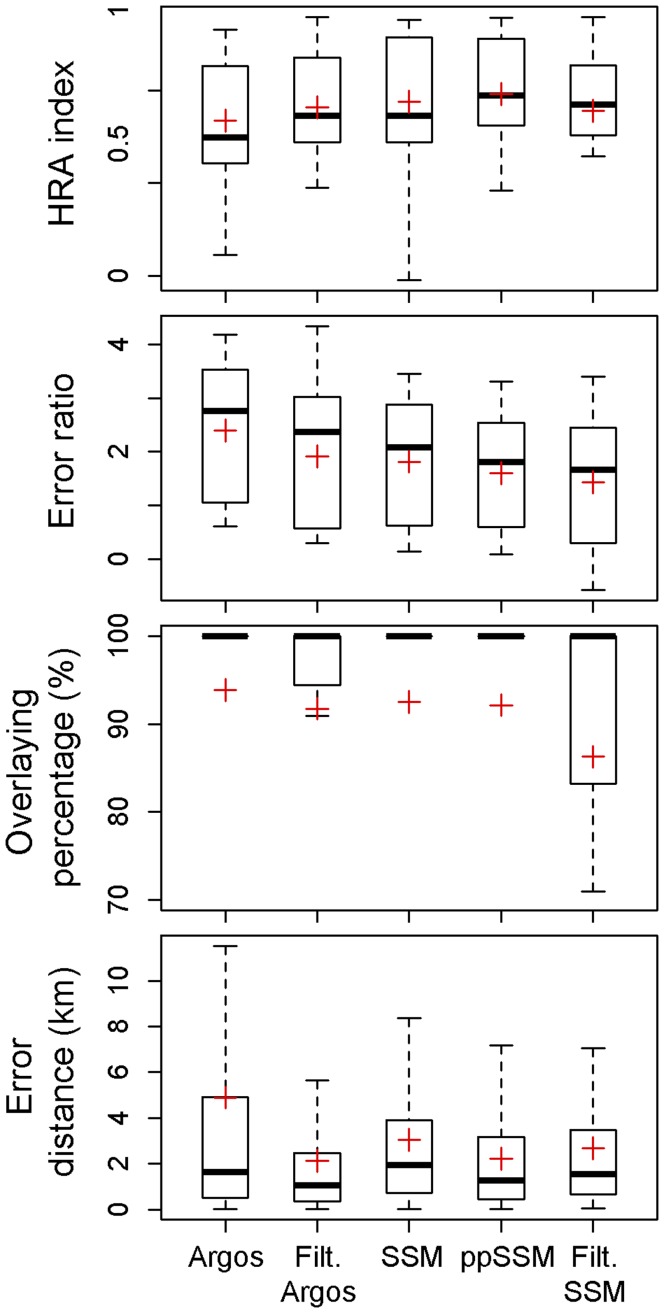
Box and whisker plots of the Home Range Accuracy (HRA) index, error ratio (log_10_ transformed), overlaying percentage (%), and error distance (km) computed using five Argos-derived location estimates. Red crosses indicate mean values. ppSSM – post-processed state-space model. Outliers are not represented.

**Figure 6:**
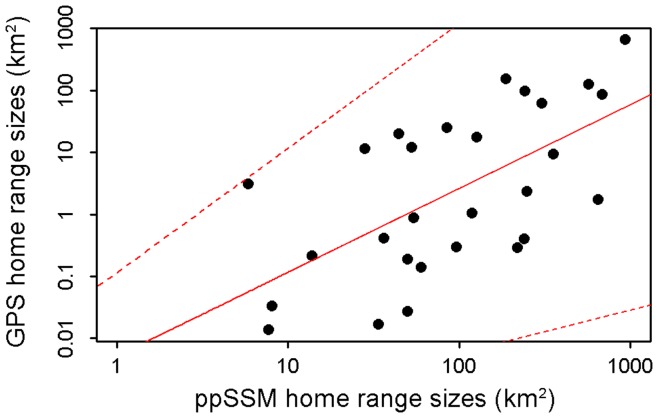
Logarithmic relationship between post-processed SSM and GPS home range sizes. The red solid line represents the best fit of a linear generalised linear model (a = 1.352, b = −2.287, adjusted r^2^ = 0.36), which had the most support (wAIC_c_  = 0.66) amongst other polynomial candidate models. Red dashed lines represent the 2.5 and 97.5% confidence intervals.

Individual habitat use analyses revealed that post-processed SSM locations best estimated the 50 and 95% UD (mean HRA index  = 0.657 and 0.718 respectively) (Table 3, Figure 5). Using this approach, home range sizes were over seven times more accurate than Argos home ranges (mean ER for 50% UD = 376.0 and 2712.0 respectively) and twice more than SSM’s (mean ER for 50% UD = 648.0) (Table 3). Filtered SSM locations produced the most accurate home range size estimates (mean ER for 50% UD = 348.5), but were associated with the lowest overlapping percentages (average OP = 82.1%) (Table 3, Figure 5). Poor overlap (<50%) was nevertheless only obtained when the number of locations for home range analyses was low (<30). Although post-processed SSM locations best recreated individual habitat use, large error ratios were obtained (Table 3, Figure 5). Such overestimation in home range size was particularly associated with spatially restricted GPS areas (median size of GPS area  = 2.0 km^2^, range  = 0.01–661.3 km^2^) (Figure 6). Animals with home ranges smaller than 3 km^2^ had a mean ER of 527.5 while those with areas larger than 3 km^2^ had a mean ER of 6.4. A linear generalised linear model best described the relationship between post-processed SSM and GPS home range sizes as it had the highest level of support (wAICc  = 0.66) and explained 38.5% of the deviance observed (Figure 6). The best fit of this linear model suggests an approximate two order of magnitude difference between post-processed SSM and GPS home range sizes for small GPS area (<5 km^2^), progressively decreasing to a one order of magnitude difference for larger GPS area (Figure 6).

**Figure 7:**
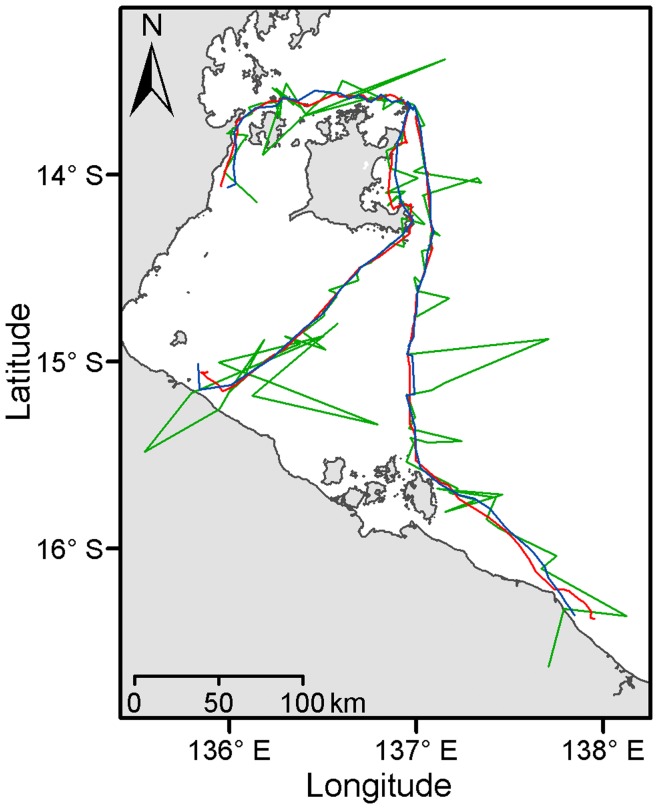
Migration tracks of three female hawksbill turtles using Fastloc GPS (red), post-processed SSM (blue) and Argos (green) locations.

Using Argos locations to recreate animal migratory pathways produced the highest errors (mean ± SD = 4.9±9.2 km, max = 77.3 km, n = 477 locations) (Figure 5 and 7). Post-processing SSM predicted locations and filtering Argos locations both minimised the distance to GPS tracks however the latter analytical approach showed a broader dispersion and fewer observations (mean ± SD = 2.2±2.4 km, max = 12.8 km, n = 399 locations and mean ± SD = 2.1±3.1 km, max = 26.2 km, n = 297 locations respectively) (Figure 5 and 7). While state-space modelling Argos data improved their accuracy (mean ± SD = 3.0±3.4 km), the same procedure applied to filtered Argos data only slightly further reduced error distances and discarded numerous fixes (mean ± SD = 2.7±3.4 km, n = 78 locations, *i.e.* ∼11 positions per individual migratory track) (Figure 5).

## Discussion

In concordance with other marine turtle studies, our Argos data were characterised by low numbers of daily uplinks and high proportions of LC 0, A, B and Z location estimates [10], [46]. Such limited transmission performances are most likely due to restricted coverage of the tropics by polar-orbiting satellites, combined with infrequent surface intervals [64], [65], [66]. Argos 68^th^ percentile LC errors were consistent with previous research, with LC 1, 2, and 3 within 1 km of “true” positions while LC ≤0 were markedly less accurate (>4 km) [15], [16]. Argos error structure was highly longitudinally skewed and was, for all LC, adequately modelled by a *t* distribution, which confirmed the non normality of Argos location error distribution [15], [17], [35], [67]. Maximum likelihood estimates of τ and ν parameters characterising *t-*distributions nonetheless differed substantially from those computed using Argos locations of caged animals [35]. While those discrepancies may be attributed to different experimental design and analytical methodology, additional research quantifying the statistical distribution parameters of Argos error is urgently required for subsequent incorporation of error probability densities into correcting algorithm.

Habitat utilisation was best quantified from post-processed SSM locations as they consistently maximised the overlap of true animal home ranges and best estimated their sizes. Argos locations greatly overestimated home range sizes and subsequent filtering of data only provided limited improvements. Applying a state-space modelling procedure on filtered Argos positions induced substantial loss in overlaying animal habitats, possibly due to low numbers of observations. The implementation of our *ad-hoc* heuristic filter thresholds discarded on average over 58.8% of Argos initial fixes (range = 18.2–89.4%), thus necessitating longer time steps for SSM analyses, resulting in the production of few predicted locations. While using Argos data in our SSMs produced home ranges with good overlap and relatively accurate sizes, post-processing those predicted locations by re-adding good Argos LC positions and removing terrestrial positions considerably refined habitat use estimation.

Our post-processing SSM approach also estimated animals’ migratory tracks with the greatest accuracy as it produced the lowest mean and maximum errors along with high numbers of observations. This post-processing procedure reduced the average distance to the GPS track by 25% compared to SSM datasets, thereby outperforming similar error assessment studies on Argos migratory tracks analysed using continuous-time SSMs (mean and median distance error of 3 and 4 km respectively) [38], [39]. The latter studies employed Kalman filters to estimate SSM parameters which necessitated prior speed filtering for Argos location errors to follow a Gaussian distribution [38], [39], [68]. Our SSM approach, on the other hand, used MCMC estimation methods, which offer additional flexibility as they allow for the incorporation of non-normal error structure. Quantifying the error associated with those two methods (maximum likelihood- vs. Bayesian estimated- SSM locations) on similar datasets nonetheless remains essential since SSM outcomes intrinsically rely on the quality of Argos data. Such assessment is particularly paramount as Service Argos now offers a new Interacting Multiple Model (IMM) algorithm using Kalman filters, which provides more locations (0.3 to 12.7% increase) with better accuracy (reduction of mean error from 10 to 65%) and less error dispersion (14 to 83% decrease) [19]. Comparative works are therefore required concomitantly to the development of new analytical procedures to highlight the most accurate processing approach for typical quantification methods of animal behaviour. We encourage those studies to use our HRA index for objective discrimination of Argos processing approaches and optimal refinement of home range estimates in exploratory analysis (*e.g.* incremental filtering).

Post-processed SSM locations benefit from the integration of Argos LC error structures into correlated random walk models and from subsequent objective filtering of biologically irrelevant, terrestrial locations. This approach can be automated and applied routinely as users only have to choose appropriate time steps and MCMC parameters. Predicted datasets are temporally regular and therefore well suited for home range analysis using KDE methods [69]. This temporal regularisation nevertheless discards small numbers of accurate Argos fixes (<10% for this study) by predicting locations at fixed time steps. Integrating those few good Argos LC positions back into SSM predicted datasets therefore provided additional information on animals’ true positions and improved both migratory track and habitat utilisation estimates. Poor overlap of true animal habitats was only observed for small sample sizes, which confirms that kernel computation requires a minimum number of observations [70], [71]. The number of locations is therefore crucial to estimate animal utilisation distribution accurately and should ideally be standardised for behavioural inference and spatial use comparisons between individuals [72]. While KDE methods don’t account for physical boundaries and may include areas of little use, they robustly describe habitat use and are more accurate than home ranges estimated from minimum convex polygon approaches [73], [74], [75]. Although we recommend future comparative studies to use the same analytical method for estimating utilisation distribution, the emergence of more complex algorithms (*e.g.* mechanistic home range models) may provide more insights into animal behaviour as they incorporate the location of external natural features (*i.e.* resources, habitat types) [76], [77], [78].

Our home range size estimates were characterised by large error ratios, especially for animals living in spatially restricted habitats as indicated by the positive linear relationship between post-processed SSM and GPS home range sizes. Our results consequently stand in contrast with the average error ratio of 2.8 (range = 1.2–3.5, n = 5 individuals) obtained for 50% UD polygon computed using azimuth filtered Argos locations [29]. The latter study nonetheless employed different kernel density estimation methods and animals displayed broader movements (GPS 50% UD area = 0.7–2.6 km^2^). The large error ratios we obtained primarily for small GPS areas may be explained by the inherent error structure of Argos data that disperses locations around animal true positions. For instance, animals inhabiting a 0.01 km^2^ area will have an estimated home range at least 400 times larger due to the average distance error of 2 km. The Fastloc GPS technology is thus preferable to investigate the fine scale spatial behaviour of species with restricted habitats as even the most parsimonious Argos data processing approach will lead to significant overestimation.

### Conclusions

Recreating animals’ paths from inaccurate data has now become an important discipline in ecology, incorporating state of the art mathematical models into complex statistical frameworks. This study constitutes an important stepping stone for wildlife tracking research as it identified the best analytical technique for processing remotely sensed Argos tracking data. Although post-processed SSM locations are still associated with higher spatial errors than Argos LC 1, 2, and 3, they provide substantial improvement for home range and migratory pathway estimation compared to Argos or filtered Argos data and consistently recreated animal spatial utilisation with the greatest accuracy amongst the set of commonly used Argos analytical methods we tested. Historical Argos datasets (*i.e.* obtained using a non-linear least-squares algorithm) can therefore be reworked using our approach to refine our knowledge of animal behaviour and provide evidence-based conservation recommendations to underpin various management strategies including protected areas. Further research is nonetheless needed as those results rely on a small number of individuals, which relayed low numbers of daily uplinks and high proportions of poor LC locations.

## Supporting Information

Table S1 **Summary of transmitter performances.**
(DOCX)
