# Supplementary material for: Enhancing the Use of Argos Satellite Data for Home Range and Long Distance Migration Studies of Marine Animals
Source: PLoS One. 2012 Jul 12;7(7):e40713. doi: 10.1371/journal.pone.0040713 (PMC3395646; doi:10.1371/journal.pone.0040713)
Supplement: Table S1 — Summary of transmitter performances. (DOCX) [file pone.0040713.s001.docx]

|  | Fastloc GPS locations | | Argos locations | | |
| --- | --- | --- | --- | --- | --- |
| Turtle | Tracking duration | Number of locations | Tracking duration | Number of locations | Proportion of LC > 0 |
| F1 | 156.9 | 631.0 | 127.9 | 358.0 | 10.1 |
| F2 | 252.9 | 979.0 | 339.1 | 931.0 | 4.8 |
| F3 | 420.7 | 1507.0 | 450.0 | 1659.0 | 23.1 |
| F4 | 119.0 | 533.0 | 170.6 | 406.0 | 14.0 |
| F5 | 85.8 | 305.0 | 121.6 | 202.0 | 5.0 |
| F6 | 85.1 | 207.0 | 73.3 | 98.0 | 8.2 |
| F7 | 83.2 | 280.0 | 140.3 | 205.0 | 1.0 |

**Table S1.** Summary of transmitter performances
